# Predictors of stroke outcome: the role of hemorheology, natural anticoagulants, and serum albumin

**DOI:** 10.1186/s41983-018-0019-x

**Published:** 2018-07-02

**Authors:** Saher S. Hashem, Sadek M. Helmy, Nervana M. El-Fayomy, Mohammed I. Oraby, Mohammed Menshawy, Nermin A. Dawood, Heba S. Hashem

**Affiliations:** 1 Neurology Department, Cairo University, Cairo, Egypt; 2 Neurology Department, Beni-Suef University, Beni-Suef, 62511 Egypt; 3 Clinical Pathology Department, Cairo University, Cairo, Egypt; 4 Neurology Department, National Institute of Research, Giza, Egypt

**Keywords:** Stroke outcome, Hemorheology, Serum albumin, Natural anticoagulants

## Abstract

**Background:**

The early hours after an acute stroke are crucial; early accurate prediction of outcome in stroke patients can help health system providers and families to choose appropriate lines of management and plan for the future. The aim of this work is to assess the role of hemorheological parameters (such as blood viscosity, hematocrit, platelet aggregation, and leukocyte count), protein C, protein S, antithrombin III, and serum albumin as predictors of stroke outcome.

**Methods:**

Thirty subjects, 20 patients with acute ischemic stroke within 24 h from the onset and 10 normal subjects, were included in this case control study. Clinical, functional, and radiological evaluation was done for the patients, and all patients and control were subjected to routine laboratory tests and assessment of blood viscosity, hematocrit level, platelet aggregation, protein C, protein S, and antithrombin III.

**Results:**

Platelet aggregation was significantly higher and serum albumin was significantly lower in stroke patients compared to control (*p* value = 0.000 and 0.039) respectively. On comparing between patient with good and poor outcome, good outcome was associated with increased serum albumin level at admission (*p* value = 0.03) respectively. A significant negative correlation was found between total leukocyte count, hematocrit value, and stroke outcome (*p* value = 0.015 and 0.013) respectively. Only albumin was found to be a significant predictor for outcome by linear regression analysis.

**Conclusion:**

Serum albumin, hematocrit level, and total leukocyte count at the time of presentation of ischemic stroke are useful markers for stroke outcome.

## Background

Given the high prevalence, disability rate, and mortality of acute ischemic stroke, prognosis has been closely evaluated. Accurate prediction of stroke outcome is of great value in setting realistic therapeutic goals, selecting proper management strategies, predicting the need for community support, and improving discharge planning (Jin et al. 2016).

Albumin is multifunctional unique serum protein which has a neuroprotective effect in different mechanisms. Albumin plays an important role as antioxidant. It was hypothesized that relatively high serum albumin is associated with good outcome in patients with acute ischemic stroke (Tomasz et al. 2004).

Acute ischemic stroke patients have marked base line hemorheology abnormalities. Important hemorheological parameters include hematocrit (Htc), blood viscosity, leukocyte counts, and platelet aggregation (Lip et al. 2002). Hematocrit plays an important role in blood oxygen carrying capacity and blood viscosity. At high levels of hematocrit, central nervous system oxygen transport diminishes as a result of increased blood viscosity and decreased cerebral blood flow (Diamond et al. 2003).

Although many studies have elaborated the role of leukocyte and neutrophil counts as independent risk factors for acute myocardial infarction and acute cerebral infarction, still data on the relationship between leukocyte count and outcome after acute ischemic stroke are inadequate (Wu et al. 2013). Also, Deficiencies of natural anticoagulants lead to tendency towards venous thromboembolism. The implication of these defects in arterial thromboses has a lot of debate and still under investigation in the recent years (Soare and Popa 2010).

## Methods

The aim of this work is to assess the role of some important hemorheological parameters (such as blood viscosity hematocrit, platelet aggregation, and leukocyte count), protein C, protein S, antithrombin III, and serum albumin as prognostic factors which can early predict the outcome after ischemic stroke.

The present study is a case control study performed in neurology inpatient ward of New Kasr El-Aini Teaching Hospital and included 30 subjects, 20 patients (13 males and 7 females) with acute ischemic stroke and 10 healthy volunteers (6 males and 4 females) matched in age and sex to stroke patients as a control. A written informed consent was obtained from each participant in this study or from one of his family members, and the study was approved by local ethical committee in Faculty of medicine, Cairo University in 28-9-2010.

Inclusion criteria were as follows: patients with first ever ischemic stroke proved by history, examination and imaging, presented within 24 h from onset and above the age of 45 years.

Exclusion criteria were as follows: hemorrhagic stroke, presentation more than 24 h from onset of stroke, recurrent stroke, cardio-embolic causes of stroke, carotid stenosis > 50% or significant non atherosclerotic disease, e.g. dissection. Patients with disturbed conscious level, diabetic patients, hypertensive patients, pregnant females or on contraceptive pills, patients with renal, hepatic or collagen vascular diseases, hyperlipidemia and hyperuricemia and smoking.

Participants of this study were subjected to the following battery of assessment during the first 24 h of stroke onset and after 1 month.

### Stage 1: During first 24 h

#### Clinical assessment

Patients were submitted to clinical evaluation including detailed history taking, general medical examination including a cardiological assessment, neurovascular examination according to the cerebrovascular stroke assessment sheet of Neurology Department, Cairo University, and level of functional impairment using the National Institute of Health Stroke Scale (NIHSS) (NIH Stroke Scale n.d.) and the Modified Rankin Scale (mRS) (Bonita and Beaglehole 1988).

Stroke severity assessed by NIHSS was categorized as mild (score 0–5), moderate (score 6–13), or severe (score ≥14) (NIH Stroke Scale n.d.).

#### Laboratory assessment

Patients and control groups were subjected to the following laboratory investigations: routine investigations (fasting and post-prandial blood sugar, complete blood count including hematocrit and ESR, serum electrolytes, serum uric acid, kidney and liver function tests, and lipid profile), protein C, S, and antithrombin III assay, assessment of blood viscosity and platelet aggregation test.

The hematological and biochemical investigations were carried out in the hematology and biochemistry units of laboratory Department of New Kasr Al-Aini Teaching Hospital, using standard commercial reagent kits.

Just after admission and before starting any intravenous infusion, blood samples from anticubital vein were collected without producing venous stasis with a 20-gauge needle in plastic disposable syringes and immediately sent to the laboratory for evaluation.

The measurement of blood viscosity was done using an Ostwald viscometer, which is a glass capillary viscometers consists of a U-shaped glass tube (Rosencranz and Bogen 2006).

The platelet aggregation test was done using Chrono-Log Lumi-aggregometer equipment, USA, which measures the optical density (turbidity) of platelet-rich plasma. Six milliliters of citrated blood samples were collected and left at room temperature for 30 min and then centrifuged at 100*g* for 10 min to obtain platelet-rich plasma.

Different substances called agonists including adenosine diphosphate (ADP), epinephrine, arachidonic acid, collagen, and thrombin are used in the test. In this study, platelet aggregation assessment was done using ADP which is lyophilized preparation of adenosine-5′-diphosphte by Bio/Data Corporation kits.

The addition of ATP to a plasma sample leads to platelets aggregation, which makes the fluid more transparent. The aggregometer then measures the increased light transmission through the specimen. The expected value ranges from 70 to 100% of norm (Ghosh et al. 2003). Antiplatelet drugs were started only after collecting the required blood samples.

#### Radiological assessment

Brain CT using Toshiba multislice 16 computed tomography, Japan, and MRI using Philips Achieva 1.5-T machine, Netherland, were performed for all patients; hemorrhagic stroke and old stroke cases were excluded. Site of infarction was classified into anterior circulation and posterior circulation and size of infarction was determined by the largest diameter of the lesion according to Shin et al. (2006).

B-mode and color-coded duplex sonography of the extracranial vessels (carotid and vertebral arteries) were performed for all patients using Philips HDI 5000 ultrasound equipment, China. Patients with carotid stenosis > 50% or significant non atherosclerotic disease were excluded from the study.

Echocardiography: transthoracic echo-Doppler study using 2D and M mode was performed for all patients using GE Vivid S5 machine, China. Patients with cardio-embolic causes of stroke were excluded from the study.

### Stage 2: After 1 month

Level of impairment using the National Institute of Health Stroke Scale (NIHSS) and the Modified Rankin Scale (mRS) were done to all patients included in this study to assess functional outcome.

Outcome was assessed by Modified Rankin Scale (mRS) after 1 month and categorized as good (mRS 0 to 2; independent) or poor (mRS 3 to 6; dependent or dead) and by the difference in NIHSS (baseline—1 month) according to Askiel et al. (2009).

#### Statistical analysis

Data was entered to Excel 2007 (Microsoft Corporation, NY, USA) and analyzed using SPSS version 19 (Statistical Package for the Social Science; SPSS Inc., Chicago, IL, USA). Data was summarized according to the type of variable. Mean and standard deviations were used to summarize quantitative variables. Frequency and proportions were used to summarize qualitative variables. Chi-square and Mann Whitney *U* test for independent samples were used to compare between groups. Simple linear regression was used to identify predictors of stroke outcome. *P* value equal to or less than 0.05 was considered significant.

## Results

This case control study included 30 subjects, 20 patients with acute ischemic stroke and 10 healthy volunteers as a control. The age of patients ranged from 46 to 80 years with a mean of 65.1 ± 7.49 years while the age of control ranged from 60 to 78 years with mean of 65.35 ± 9.29 years. Patient subjects were 13 males (65%) and 7 females (35%) while control subjects were 6 males (60%) and 4 females (40%).

The clinical assessment scales which were done for stroke patient at admission and after 1 month are summarized in Table 1. According to the results of NIHSS and mRS scores after 1 month, 12 (60%) patients were classified as having good outcome (mild severity in NIHSS score, or grade 1 and 2 in mRS score) and 8 (40%) patients with poor outcome (moderate severity in NIHSS score, or grade 3 in mRS score) (Rosencranz and Bogen 2006; Bhatia et al. 2004) (Table 2).

**Table 1:** NIHSS and mRS scores on admission and after 1 month

	Mean	± S.D	Minimum	Maximum
mRS on admission	4.20	0.89	3	6
mRS after 1 month	2.35	0.59	1	3
NIHSS on admission	8.65	2.54	5	14
NIHSS after 1 month	4.95	2.87	2	12
NIHSS difference	3.70	0.92	2	5

*mRS* Modified Rankin Scale, *NIHSS* National Institute of Health Stroke Scale

**Table 2:** Stroke outcome after 1 month according to NIHS and mRS scores

	NIHSS			mRS		
	Score	No.	Percent	Score	No.	Percent
Good outcome	0 to 5	12	60	1	1	5
				2	11	55
Bad outcome	6 or more	8	40	3	8	40

*mRS* Modified Rankin Scale, *NIHSS* National Institute of Health Stroke Scale

In comparing patients and control groups regarding results of laboratory investigations, there was significant difference between patients and control groups regarding platelet aggregation (being more in patients group) and serum albumin (being more in control group), *p* value 0.000 and 0.039 respectively (Table 3).

**Table 3:** Comparison between patients and control group regarding results of laboratory investigations

	Subjects	No.	Mean	± S.D	*p* value
Antithrombin III	Cases	20	98.73	17.98	0.628
	Controls	10	102.11	17.55	
Protein S	Cases	20	99.04	20.76	0.515
	Control	10	104.30	20.29	
Protein C	Cases	20	107.10	18.19	0.949
	Control	10	106.60	23.63	
Platelet aggregation	Cases	20	94.54	5.79	0.000**
	Controls	10	82.50	7.49	
Blood viscosity	Cases	20	1.70	0.16	0.790
	Control	10	1.71	0.10	
Total leukocyte count	Cases	20	7.91	2.10	0.353
	Controls	10	7.21	1.45	
Hematocrit	Cases	20	39.71	4.13	0.995
	Control	10	39.70	2.80	
Albumin	Cases	20	3.58	0.54	0.039*
	Controls	10	3.98	0.35	

*p value is significant at the 0.05 level

**p value is significant at the 0.01 level (highly significant)

Comparison was carried between patient with good outcome (12 patients) and poor outcome (8 patients) as regard laboratory investigations (Table 4). There was a significant difference between the two groups only regarding serum albumin (being more in patients with good outcome), *p* value 0.030.

**Table 4:** Comparison between patient with good and poor outcome as regard laboratory investigations

	Outcome	No.	Mean	± S.D	*p* value
Antithrombin III	Good	12	96.58	14.78	0.643
	Poor	8	101.95	22.69	
Protein S	Good	12	101.23	19.45	0.395
	Poor	8	95.75	23.55	
Protein C	Good	12	100.42	15.17	0.034
	Poor	8	117.13	18.59	
Platelet aggregation	Good	12	93.98	5.67	0.969
	Poor	8	95.38	6.25	
Blood viscosity	Good	12	1.66	0.09	0.261
	Poor	8	1.75	0.23	
Total leukocyte count	Good	12	7.28	1.93	0.089
	Poor	8	8.86	2.09	
Hematocrit	Good	12	38.78	4.41	0.070
	Poor	8	41.11	3.45	
Albumin	Good	12	3.80	0.56	0.030 *
	Poor	8	3.24	0.26	

*p value is significant at the 0.05 level

Correlation was carried between size of infarction and results of laboratory investigation. There was significant positive correlations between blood viscosity and size of infarction (*r* = 0.491, *p* value = 0.028) Table 5.

**Table 5:** Correlation between size of infarction and laboratory results

	Size of infarction	
	Correlation coefficient (*r*)	Probability value (*p* value)
Antithrombin III	0.240	0.308
Protein S	0.120	0.614
Protein C	− 0.360	0.119
Platelet aggregation	− 0.103	0.665
Blood viscosity	0.491	0.028*
Total leukocyte count	− 0.138	0.562
Hct	− 0.176	0.459
Albumin	0.076	0.749

*Correlation is significant at the 0.05 level

Correlation was carried between stroke outcome (measured by difference in NIHSS on admission and NIHSS after 1 month) and results of laboratory investigation. There was a high significant positive correlation between albumin and stroke outcome (*r* = 0.632, *p* value = 0.003), a significant negative correlation between total leukocyte count and stroke outcome (*r* = − 0.536, *p* value = 0.015) and significant negative correlations between hematocrit and stroke outcome (*r* = − 0.545, *p* value = 0.013) (Table 6).

**Table 6:** Correlation between stroke outcome and results of laboratory investigation

	NIHSS difference	
	Correlation coefficient (*r*)	Probability value (*p* value)
Antithrombin III	− 0.188	0.428
Protein S	− 0.003	0.991
Protein C	− .321	0.168
Platelet aggregation	− 0.362	0.117
Blood viscosity	0.060	0.800
Total leukocyte count	− 0.536*	0.015*
Hematocrit	− 0.545*	0.013*
Albumin	0.632**	0.003**

*Correlation is significant at the 0.05 level

**Correlation is significant at the 0.01 level (highly significant)

There were no significant correlations between antithrombin III, protein S, protein C, platelet aggregation or blood viscosity, and stroke outcome Table 6.

### Regression analysis

**Fig. 1:**
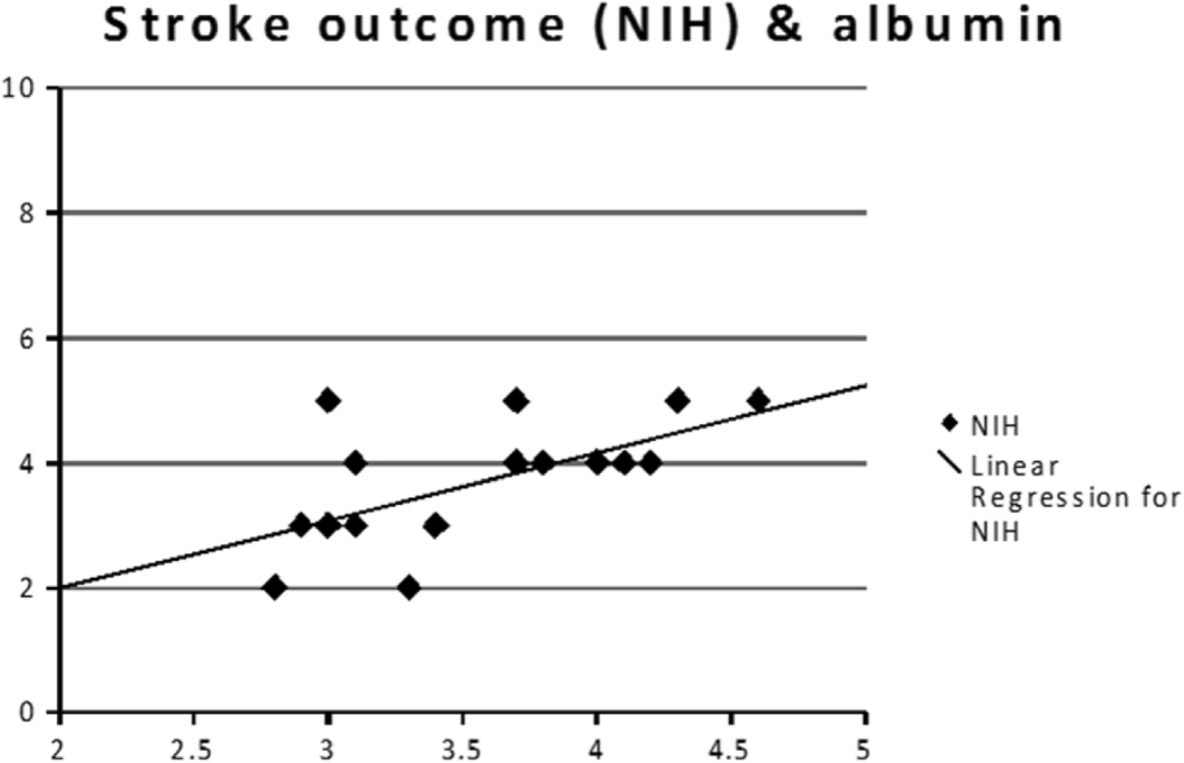
Correlation between serum albumin and stroke outcome

Linear regression analysis was done to test for significant predictors of outcome (measured by difference in NIHSS on admission and NIHSS after 1 month). Antithrombin III, protein C, protein S, platelet aggregation, blood viscosity, hematocrit, total leukocyte count, and albumin were entered in regression model, but only albumin was found to be significant predictor for outcome Fig. 1.

## Discussion

The early hours after an acute stroke are crucial; it is the most useful time for effective management. With the rising burden of stroke and marked heterogeneity in stroke manifestation and outcome, it is necessary to find accurate, reliable, and simple predictors of functional recovery. Also, the natural recovery patterns must be investigated more so that we can better assess the effectiveness of the available therapeutic interventions and their contribution to recovery process (Bhatia et al. 2004).

In this study, we assessed the role of some important hemorheological parameters (such as blood viscosity hematocrit (Htc), platelet aggregation, and leukocyte count), protein C, protein S, antithrombin III, and serum albumin as prognostic factors which can early predict the outcome after ischemic stroke.

Albumin is an important functional protein in the blood; it maintains the normal permeability of the microvessel wall, reduces blood viscosity, and inhibits platelet aggregation (Wei-Hai et al. 2014). In recent years, there has been increasing interest in the association between serum albumin levels and stroke. Many studies have demonstrated an inverse relation between the concentration of serum albumin, stroke risk, and functional outcome (Babu et al. 2013).

Our results revealed that the serum albumin at admission is an independent predictive factor of the functional outcome in ischemic stroke patients by linear regression statistical analysis which is done to test for significant predictors of outcome. It suggests that patients with low serum albumin level within 24 h after the onset of stroke may have a poor functional outcome and it may be caused by low neuroprotective effects.

Many recent studies have shown the prognostic role of serum albumin level in cases of acute ischemic stroke. Our results agreed with Wei-Hai et al. (2014), Babu et al. (2013), Yoon et al. (2008), and Kasundra and Sood (2014) who investigated the effect of serum albumin at admission, on the functional outcome in ischemic stroke patients. They concluded that serum level of albumin at admission is considered as a good indicator of the functional outcome and trials for the correction of hypoalbuminemia in acute ischemic stroke would be helpful to decrease the risk of poor outcome.

Belayev et al. (2001) found that moderate-dose albumin therapy markedly improves functional outcome in ischemic stroke patients and reduces infarction volume and brain swelling.

High-dose albumin treatment for acute ischemic stroke was assessed in a randomized, double-blind, placebo-controlled trial, and the results were not encouraging further studies to test the role of albumin on a larger scale, at a different dose, and over a longer duration of follow-up were suggested by the authors (Ginsberg et al. 2013).

In the present study, there was a statistically significant negative correlation between hematocrit and clinical outcome. An elevated hematocrit can enhance atherosclerosis by increasing protein infiltration into the vessel wall, promoting platelet adhesion to the subendothelium and by causing stagnation of blood flow (Irace et al. 2003).

Our results agreed with Levy et al. (1986), Diamond et al. (2003), and Allport et al. (2005) who pointed out that hematocrit is inversely related to neurologic outcome in acute ischemic stroke and showed that the effect of hematocrit on outcome is not related to sex, age, diabetes, or hypertension. Czlonkowska et al. (1997) also demonstrated that hematocrit is an important predictor factor of 30-day fatality in ischemic stroke.

On the other hand, our results were opposed by Ozaita et al. (1987) who concluded that hematocrit has no effect on the short-term outcome of ischemic stroke cases. However, their findings also showed that there was only a tendency to elevated hematocrit in the first days in cases with severe admission deficit. Moreover, Bhatia et al. (2004) found that hematocrit is not a predictive factor of 30-day fatality in ischemic stroke.

The importance of the effect of hematocrit on stroke outcome explains the role of hemodilution therapies that may potentially improve stroke outcome. Previous hemodilution strategies have aimed to increase cerebral blood flow and salvage penumbral tissue. Unfortunately, early hemodilution trials in management of human acute ischemic stroke were negative, and the failure of translation from the trials of animal models has been attributed to different time windows and methodologic limitations in human (Asplund 2004).

Leukocyte count has an established role in predicting incident cerebrovascular and cardiovascular diseases, but the relationship between leukocyte count and acute ischemic stroke outcome is not well investigated (Elkind et al. 2004). In this study, a statistically significant negative correlation was found between total leukocyte count and clinical outcome (*p* value = 0.015).

Our results agreed with many recent studies; Furlan et al. (2014) in their study which was conducted on 170 cases with acute ischemic stroke found that the risk of stroke leading to disability at discharge was 1.04 times greater for every increase of 1 × 10^9^ in leukocyte count on admission. Also, survival curves showed that high leukocyte count was associated with worsening in the 30-day mortality in patients with acute ischemic stroke.

Also, Liang et al. (2016) in their study which included 8829 patients with acute ischemic stroke reported that increased leukocyte on admission is an independent predictor of stroke severity on admission, higher degree of disability at discharge, and 30-day mortality.

Our study disagrees with an older study which studied the effect of leukocytosis on stroke outcome and reported that increased total leukocyte count on admission was related only to initial stroke severity but not to functional outcome (Kammersgaard et al. 1999).

Blood viscosity assumes a vital role in the pathophysiology of vascular diseases as a factor determining global cardiovascular load, and as a variable influencing regional tissue perfusion. Additionally, a high blood viscosity increases the risk of thromboembolic events and is correlated with the presence of systemic inflammation (Pop et al. 2002).

Our study found a statistically significant positive correlation between blood viscosity and size of infarction (*p*-value = 0.028) but failed to find any relation between blood viscosity and stroke outcome. This result was agreed with Zhou and Yang (1990) who reported that blood viscosity has positive correlation with the size of the cerebral infarction and postulated that the hematocrit and the blood viscosity should be reduced immediately for treating the larger cerebral infarction.

Platelet aggregation is a vital step in thrombus formation; several studies have demonstrated that platelets are activated in the acute phase of ischemic stroke. However, the impact of enhanced platelet activation in the clinical and functional outcome of acute ischemic stroke was still not well investigated. Recently, many studies have demonstrated that enhanced platelet activation is related to larger infarct size and poor clinical and functional outcome in patients with acute ischemic stroke (Zeller et al. 2005; Yip et al. 2005).

This study found a significant difference between cases and control group as regard platelet aggregation (*p* value 0.000) which agreed with Fateh-Moghadam et al. (2007) who reported that patients with acute ischemic stroke had significantly enhanced platelet aggregability compared to patients who presented with TIA. Also systemic platelet activation is enhanced in patients with acute stroke or TIA and returns to baseline levels at 3-month follow-up. Moreover, at 3-month follow-up, persistent platelet activation is associated with increased incidence of recurrent stroke.

However, in this study, there was no statistically significant correlation between platelet aggregation and clinical outcome. This finding coincided with Bhatia et al. (2004) who found that stroke outcome was not affected by platelet aggregation and there was no significant difference amongst expired patients and survivors regarding platelet aggregation. On the contrary, Iwamoto et al. (2005) concluded that enhanced platelet aggregation is associated with poor prognosis and that platelet function test helps to predict outcome in stroke patients.

The role of the natural anticoagulants, antithrombin III, protein C, and protein S, in patients with ischemic stroke, remains uncertain. This study aimed to find out whether their levels in peripheral blood correlate with the severity of neurological deficit or can predict clinical outcome and recurrence. There was no significant correlation between antithrombin III, protein C, and protein S and stroke outcome.

The patients included in this study were above 46 years old while natural anticoagulants deficiency may play a role in stroke in young adults. Additionally, natural anticoagulants deficiency is considered as one of the main risk factors of venous infarctions with less clear role in arterial infarctions.

Our results partially coincided with Haapaniemi et al. (2002) who found no correlation between the levels of the natural anticoagulants and etiology of stroke, any stroke risk factor, or neurological scores, except that the antithrombin III level on admission showed significant correlation with stroke severity and disability at 3 months. Moreover, natural anticoagulant levels did not predict recurrence of ischemic stroke.

## Conclusions

At the end of this study, we conclude that decreased serum albumin, increased hematocrit level, and increased total leukocyte count at the time of presentation of ischemic stroke are associated with less favorable outcome, while antithrombin III, protein s, protein c, platelet aggregation or blood viscosity have no clear role in stroke outcome.

The limitation of this work is the relatively small number of patients due to the limitation of resources and financial issues.

Further studies should be conducted on a larger number of patients and for a longer duration to estimate the remote outcome and to study the effect of correction of hypoalbuminemia and the effect of hemodilution therapies on stroke outcome.

## Abbreviations

ADP: Adenosine diphosphate
CT: Computed tomography
Htc: Hematocrit
MRI: Magnetic resonance imaging
mRS: Modified Rankin Scale
NIHSS: National Institute of Health Stroke Scale
